# Supplementary material for: Identifying clinically relevant cell state interactions in the tumor microenvironment of IDH-mutant gliomas using CSI-TME
Source: Mol Syst Biol. 2026 Mar 10;22(6):928–61. doi: 10.1038/s44320-026-00201-0 (PMC13230996; doi:10.1038/s44320-026-00201-0)
Supplement: Supplementary file 2 — Appendix [file 44320_2026_201_MOESM2_ESM.pdf]

Appendix for Identifying Clinically Relevant Cell State Interactions in the Tumor Microenvironment of IDH-Mutant Gliomas Using CSI-TME

Table of Contents

Independent ascertainment of the cell state interactions in CGGA cohort *2*

Appendix table S1 3

Appendix figure S1 (Related to main Figure 2)...4

Independent ascertainment of the cell state interactions in CGGA cohort

In figure 2G, cell state interactions that we identified in CGGA cohort by projecting it onto the ICA factorization space derived from TCGA cohort resulted in a significant cross-cohort reproducibility. Here we aimed to assess the extent of reproducibility between the CSIs detected interpedently in TCGA and CGGA without relying on the TCGA fitted ICA models. Towards this, we independently factorized the CGGA cohort and computed the CSIN by using our pipeline and assessed the extent to which an interaction detected in TCGA is also detected (p-value < 0.05) in CGGA. Since in this case, TCGA and CGGA ICA factorizations are independent of each other, we first need to map the ICs between two cohorts. For each cell type, we defined this mapping by computing the Pearson's correlation coefficient between the gene-weights of the ICs in TCGA and CGGA. Since each cell type had 10 ICs, this results in 100 (10 x 10) correlation values for each cell type between TCGA and CCGA. We define mapping using a correlation threshold of 0.20. We used absolute correlation (instead of signed correlation) because the signs of features in ICA mixing matrix in the two cohorts may be inverted.

Once we had the mapping between the TCGA and CGGA ICs, we ascertained the extent of reproducibility by directly overlapping the interactions detected in TCGA and CGGA while adjusting for the IC mappings and the IC directions. For instance, for two interacting TCGA cell states, IC^x and IC^y, in Bin9, suppose we have a corresponding IC pair ($IC^{x'}$ and $IC^{y'}$) in CCGA where both *x*, *x'* and y, y' are negatively correlated with each other. Due to this inverse relationship, the Bin 9 (which implies the simultaneous upregulation of IC^x and IC^y in TCGA), will map onto $IC^{x'}$ and $IC^{y'}$ in Bin.1 in CGGA. The mapping between IC pairs and their interaction bins is provided in the Appendix Table S1.

In this table IC1 and IC2 represent the pairs of ICs that interacted in Int_bin in TCGA, and Mapped_bin is the target bin in CGGA after adjusting for the signs of ICs. The values in columns IC1 and IC2 represent the direction of correlation between the ICs in TCGA and CGGA.

Following this rule, we observed that 82 / 160 (>51%) interactions in TCGA could be independently detected in CGGA cohort without relying on TCGA ICA factorization space. In contrast, we only observed a median overlap of ~7% when using 10 randomized sets of cell state interactions. This result, together with Figure 2A and EV2I, suggests a significant reproducibility between TCGA and CGGA in terms of CSIs despite their technical and genetic differences.

Appendix table S1 - A schematic table showing the interaction bin mapping (column Mapped_bin) between independently detected CSIs in TCGA and CGGA depending on the sign of correlation (columns IC1 and IC2), interaction bin (Int_bin).

IC1	IC2	Int_bin	Mapped_bin
1	1	Bin.1	Bin.1
1	1	Bin.3	Bin.3
1	1	Bin.9	Bin.9
-1	1	Bin.1	Bin.3
-1	1	Bin.3	Bin.1
-1	1	Bin.9	Bin.7
1	-1	Bin.1	Bin.7
1	-1	Bin.3	Bin.9
1	-1	Bin.9	Bin.3
-1	-1	Bin.1	Bin.9
-1	-1	Bin.3	Bin.7
-1	-1	Bin.9	Bin.1

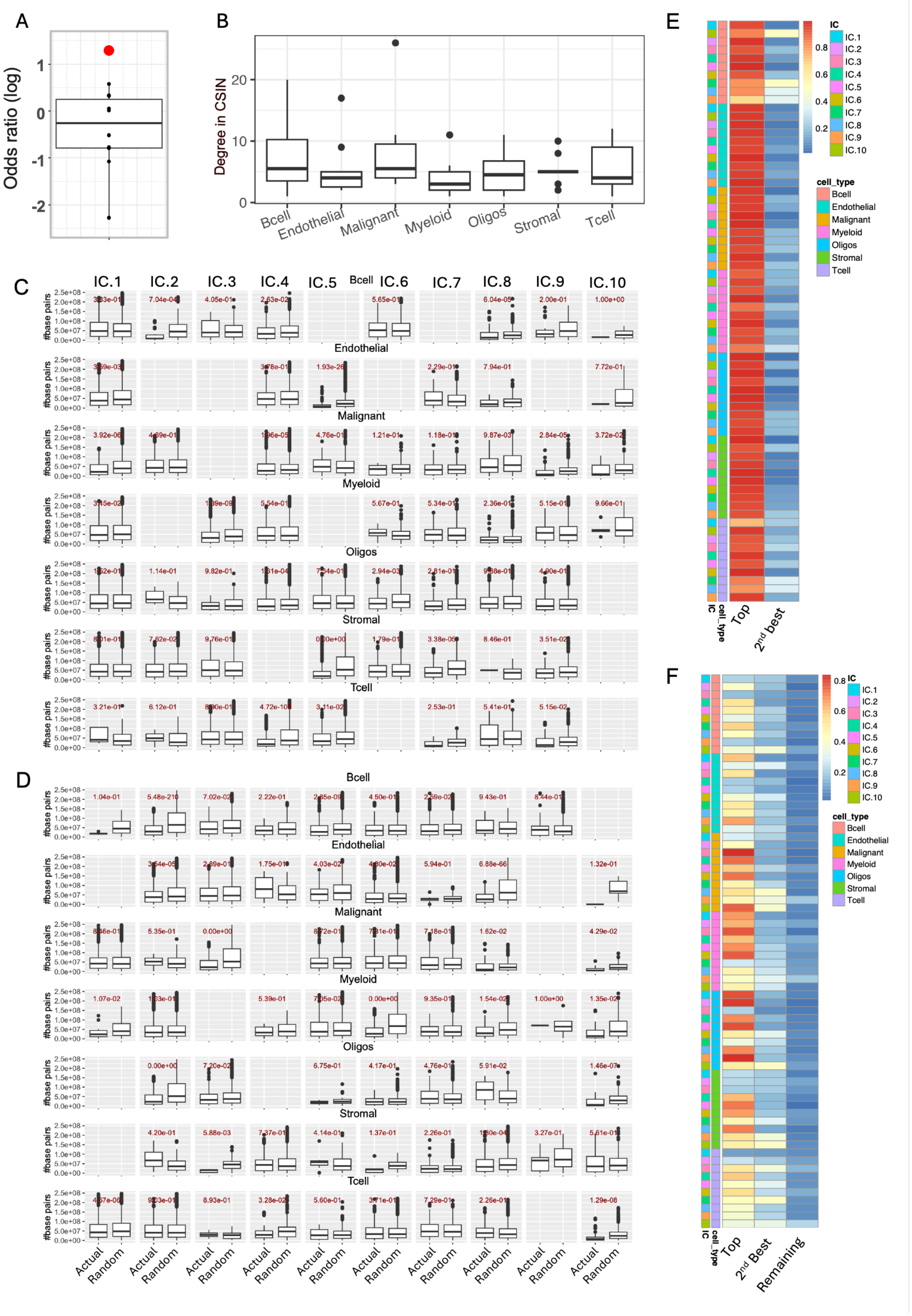

Appendix figure S1 (Related to main Figure 2) **A.** Boxplot showing the log odds ratio of enrichment for significant interactions (nominal p-value < 0.05) between TCGA and CGGA (red point) using the randomized clinical data. The odds ratio between actual interactions at a similar p-value

threshold is shown by the red dot. **B.** Boxplot showing the distribution of the number of interactions observed for the ICs of each cell type. **C.** Boxplots showing the linear clustering of the positive signature genes of various ICs. On Y axis is the distribution of pairwise linear chromosomal distances (in base-pairs) among the positive signature genes of various ICs (i.e. IC.1, IC.2 etc.) that are present on same chromosome. For each IC, the actual distribution of linear-chromosomal distances between the signature genes is compared against the distribution of genomic distances between 10 randomly drawn gene sets. These randomly drawn gene sets had a similar chromosomal distribution to the signature genes of the corresponding ICs. The ordering of ICs is provided at the top of the figure. P-values from Wilcoxon's tests are shown. **D.** Same as J but for negative signature genes. Genomic distances were calculated only for those ICs which had at least 3 signature genes with remaining cases shown as blank plots. In **A-D**, the horizonal line in the middle is the median value with lower and upper edges of the boxes corresponding to the 25th and 75th percentiles and vertical lines corresponding to 1.5 times the interquartile range. **E.** Heatmap showing average Spearman's correlation of each IC computed from full data with each IC computed from the 70% bootstrapped data in TCGA for the same cell type. The sudden drop-in second-best correlation highlights that each IC can be uniquely recovered in the bootstrapped data. **F.** Heatmap showing the Spearman's correlation of each IC computed from full data in TCGA with each IC discovered independently in the CGGA data. A significant drop-in second-best correlations and poor correlation across cell types highlight the specificity and robustness of the ICs.
