# Supplementary material for: Identifying clinically relevant cell state interactions in the tumor microenvironment of IDH-mutant gliomas using CSI-TME
Source: Mol Syst Biol. 2026 Mar 10;22(6):928–61. doi: 10.1038/s44320-026-00201-0 (PMC13230996; doi:10.1038/s44320-026-00201-0)
Supplement: Supplementary file 21 — Expanded View Figures [file 44320_2026_201_MOESM21_ESM.pdf]

Expanded View Figures

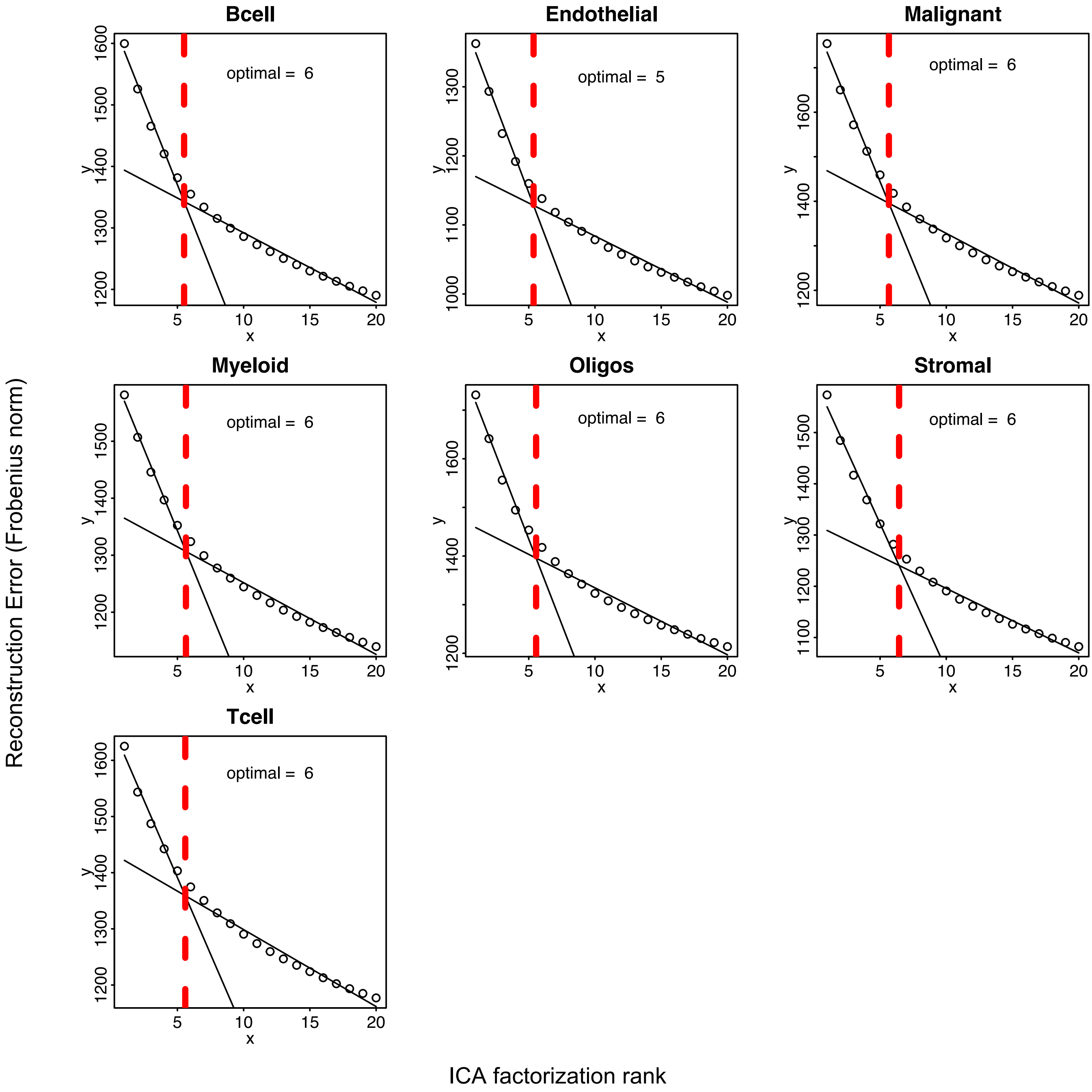

Figure EV1. Estimation of optimal rank for ICA factorization.

Each scatter plot corresponds to a specific cell type shown at the top of the plot. *X*-axis spans the ICA factorizations starting from rank = 1 up to rank = 20, and *Y*-axis is the reconstruction error computed as Frobenius norm between original gene expression matrix and reconstructed gene expression matrix using ICA factorizations of ranks ranging from 1 to 20. Source data are available online for this figure.

© This is a U.S. Government work and not under copyright protection in the US; foreign copyright protection may apply

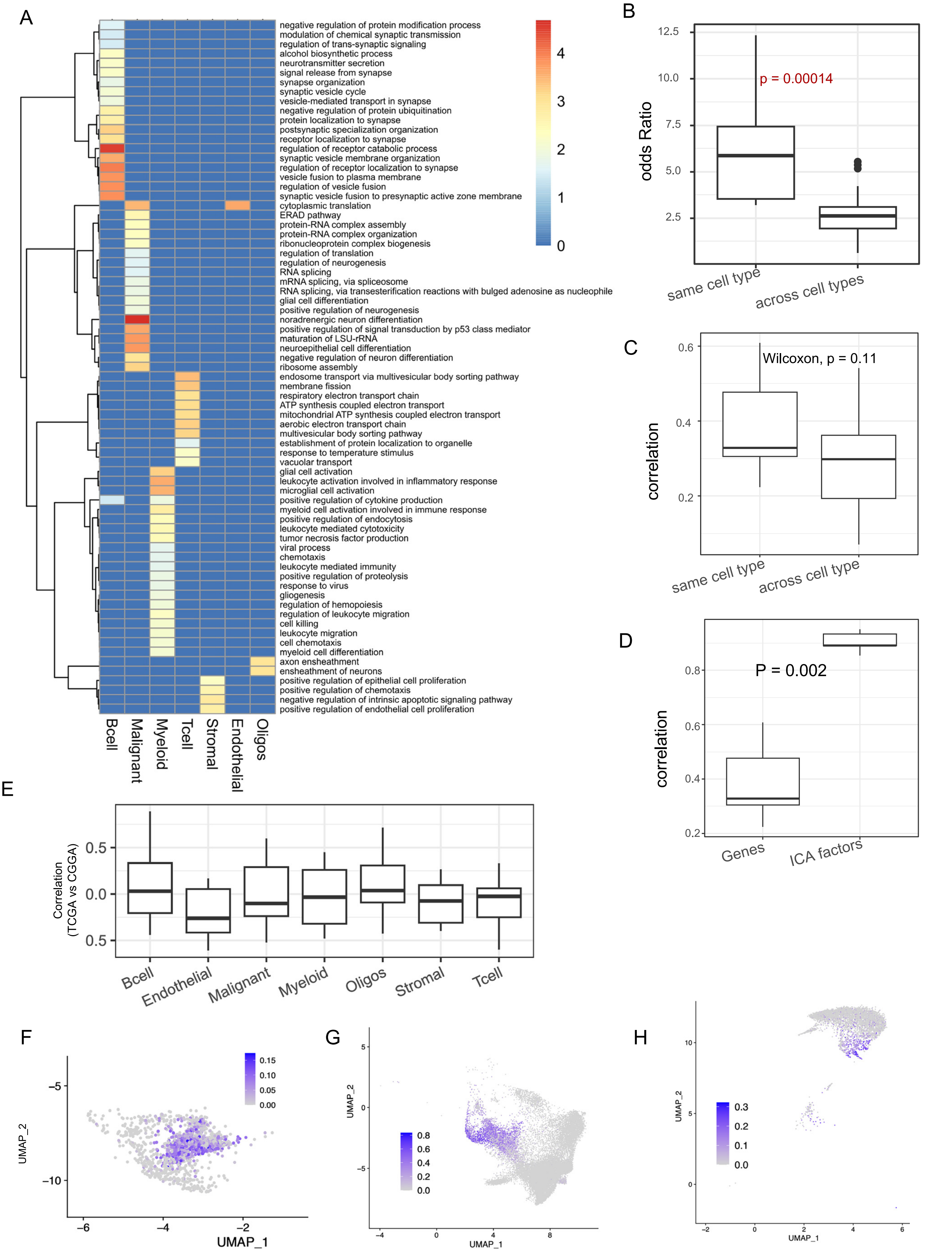

© This is a U.S. Government work and not under copyright protection in the US; foreign copyright protection may apply

Figure EV2. Assessment of the quality of deconvolution and cell state inference.

(**A**) Heatmap showing the enrichment of distinct functions among the top genes expressed by each cell type in the deconvolved data. The colors in the heatmap indicate the odds ratio of enrichment calculated as the fraction of genes belonging to the enriched functional category in the test set compared to the background set. The scale bar is provided on the right side. (**B**) Boxplot showing the odds ratio of enrichment among the top genes expressed between the same cell type and across different cell types in the deconvolved data in TCGA and CGGA (**C**). A box plot showing the cross-gene correlations (left column) of log-hazard ratio between TCGA and CGGA for the same cell type and across cell type comparisons. (**D**) Boxplot showing the distribution of correlation of hazard ratios for the same cell types between TCGA and CGGA, computed either using genes or using ICs. (**B–D**) *P* values from Wilcoxon's rank-sum test is shown. (**E**) Boxplot showing the distribution of correlation of hazard ratios for each cell type between TCGA and CGGA across 10 different randomizations of the input data. (**B–E**) The horizontal line in the middle is the median value, with lower and upper edges of the boxes corresponding to the 25th and 75th percentiles, and vertical lines corresponding to 1.5 times the interquartile range. (**F–H**) UMAP plots for T cells (**F**), malignant cells (**G**) and Oligodendrocytes (**H**) colored by the signature scores derived from the signature genes of IC1 (T cells), IC7 (Malignant cells), and IC7 (Oligodendrocytes), respectively. Source data are available online for this figure.

© This is a U.S. Government work and not under copyright protection in the US; foreign copyright protection may apply

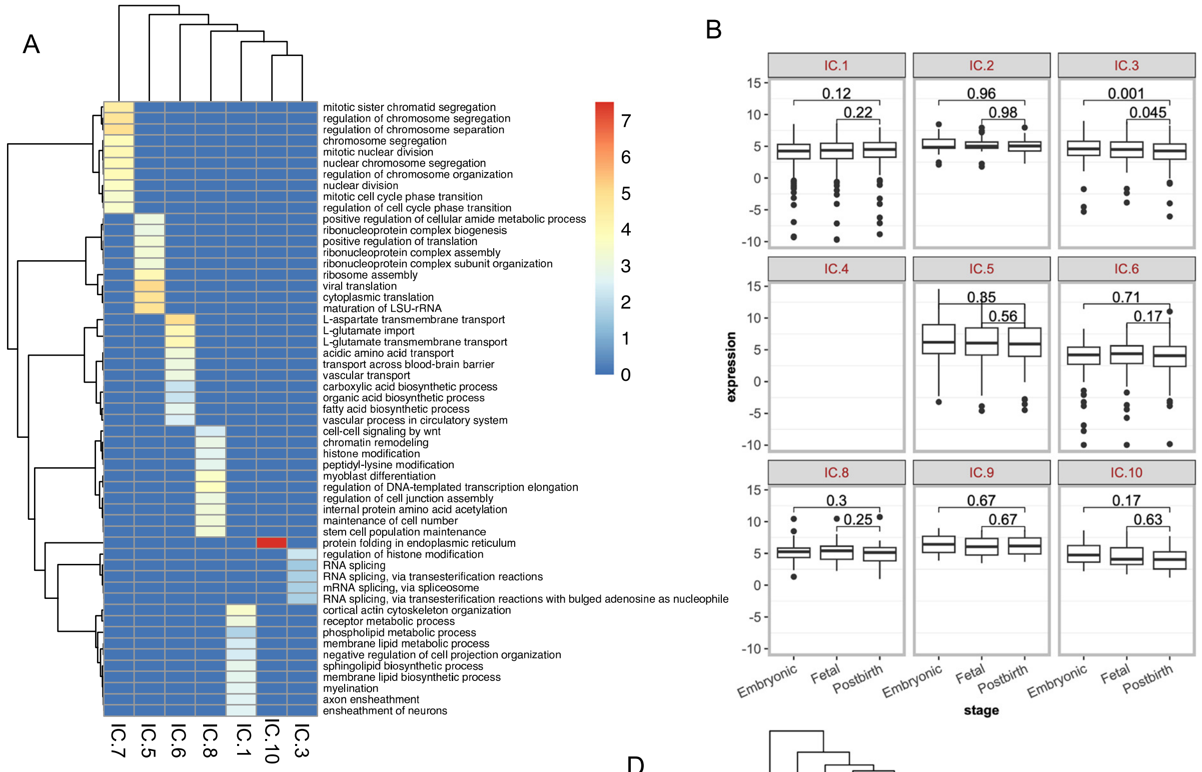

C

IC1 Direction	IC2 Direction	Interaction Bin	Activity state
Positive	Positive	Bin 1	IC1 ↓ IC2 ↓
Positive	Positive	Bin 3	IC1 ↑ IC2 ↓
Positive	Positive	Bin 9	IC1 ↑ IC2 ↑
Negative	Negative	Bin 1	IC1 ↑ IC2 ↑
Negative	Negative	Bin 3	IC1 ↓ IC2 ↑
Negative	Negative	Bin 9	IC1 ↓ IC2 ↓
Positive	Negative	Bin 1	IC1 ↓ IC2 ↑
Positive	Negative	Bin 3	IC1 ↑ IC2 ↑
Positive	Negative	Bin 9	IC1 ↑ IC2 ↓

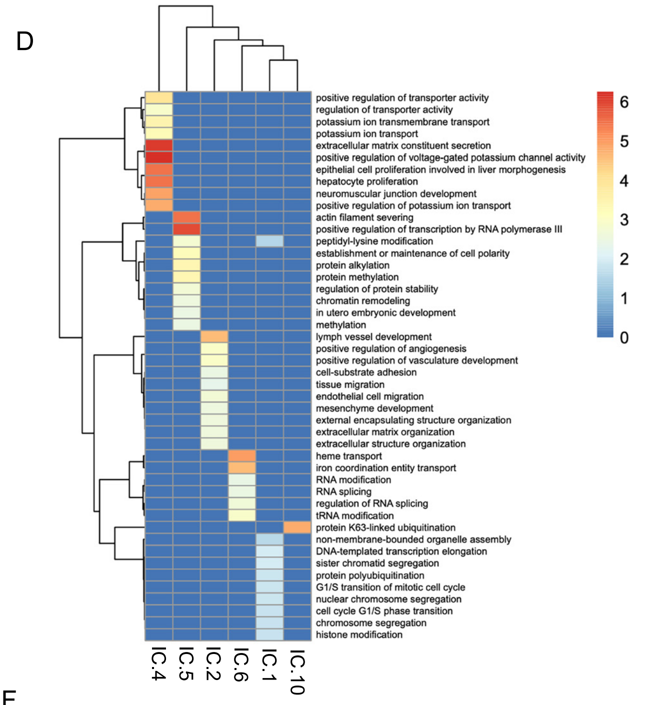

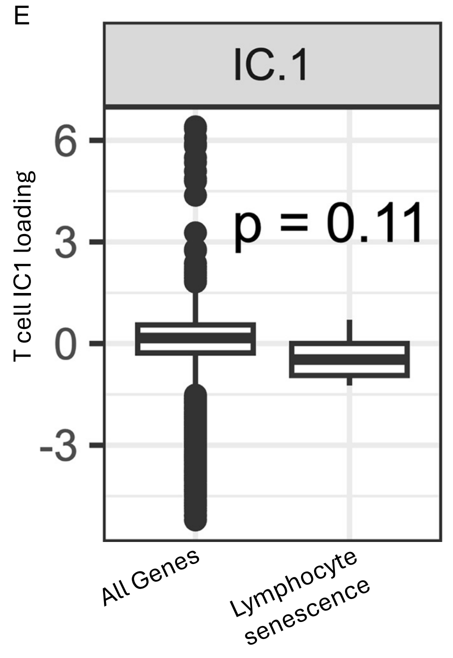

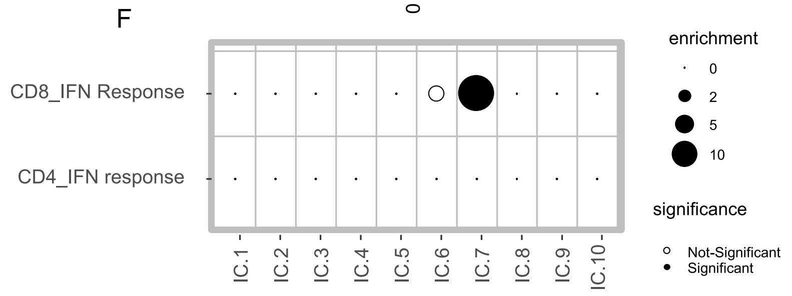

 © This is a U.S. Government work and not under copyright protection in the US; foreign copyright protection may apply

Figure EV3. Identification of glioma stem cells and their interactions with other cell types in TME.

(**A**) A heatmap showing the GO terms enriched across the negative signature genes of the Malignant cell ICs. The cell colors show the odds ratio of enrichment for each significant GO term in at least one of the comparisons. (**B**). A boxplot showing the expression (in log TPM units) of negative signature genes of various malignant cell ICs during the embryonic development of the human brain. The samples sizes (N) are as follows (IC1:233, IC2:24, IC3:277, IC4:0, IC5:106, IC6:164, IC8:58, IC:92, IC10:18) *P* values from Wilcoxon's rank-sum test are shown. The pattern for IC.7 is shown in Fig. 3D and IC.4 is shown as blank, as there were no signature genes as per our criteria (**C**). A schematic showing the activity state of positive and negative signature genes of interacting ICs across different activity bins. Arrows indicate upregulation (upwards arrow) and downregulation (downwards arrow). For instance, the signature genes at the positive end of an interacting IC pairs, say IC1 and IC2 are simultaneously downregulated in Bin 1, simultaneously upregulated in Bin 9, and exhibit upregulation of IC1 and downregulation of IC2 in Bin 3. Analogously, the signature genes at the negative end of the interacting IC pairs, say IC1 and IC2, are simultaneously upregulated in Bin 1, simultaneously downregulated in Bin 9, and exhibit downregulation of IC1 and upregulation of IC2 in Bin 3. (**D**) A heatmap showing the GO terms enriched across the negative signature genes of the T cell ICs. The cell colors show the odds ratio of enrichment for each significant GO term in at least one of the comparisons. (**E**) A box plot showing the contribution of lymphocyte-specific senescence markers ($N = 6$) to IC1 of T cells along with the genome-wide ($N = 8747$) distribution. *P* value from Wilcoxon's rank-sum test is shown. (**B**, **E**) The horizontal line in the middle is the median value with lower and upper edges of the boxes corresponding to the 25th and 75th percentiles, and vertical lines corresponding to 1.5 times the interquartile range. (**F**) A dot plot showing the odds ratio for the overlap between negative signature genes of T cells IC7 and interferon response signaling in CD8 and CD4 T cells. The size of dots is proportional to the odds ratio derived from Fisher's exact test, and *P* values were adjusted for multiple comparisons across all the curated T cell state markers provided by Yanshuo et al. In both Fig. EV2A,D, colors in the heatmap indicate the odds ratio of enrichment calculated as the fraction of genes belonging to the enriched functional category in the test set compared to the background set. The scale bar is provided on the right side. The ICs are ordered as per the dendrogram on the top and only the ICs with at least one significantly enriched function are plotted. Source data are available online for this figure.

© This is a U.S. Government work and not under copyright protection in the US; foreign copyright protection may apply

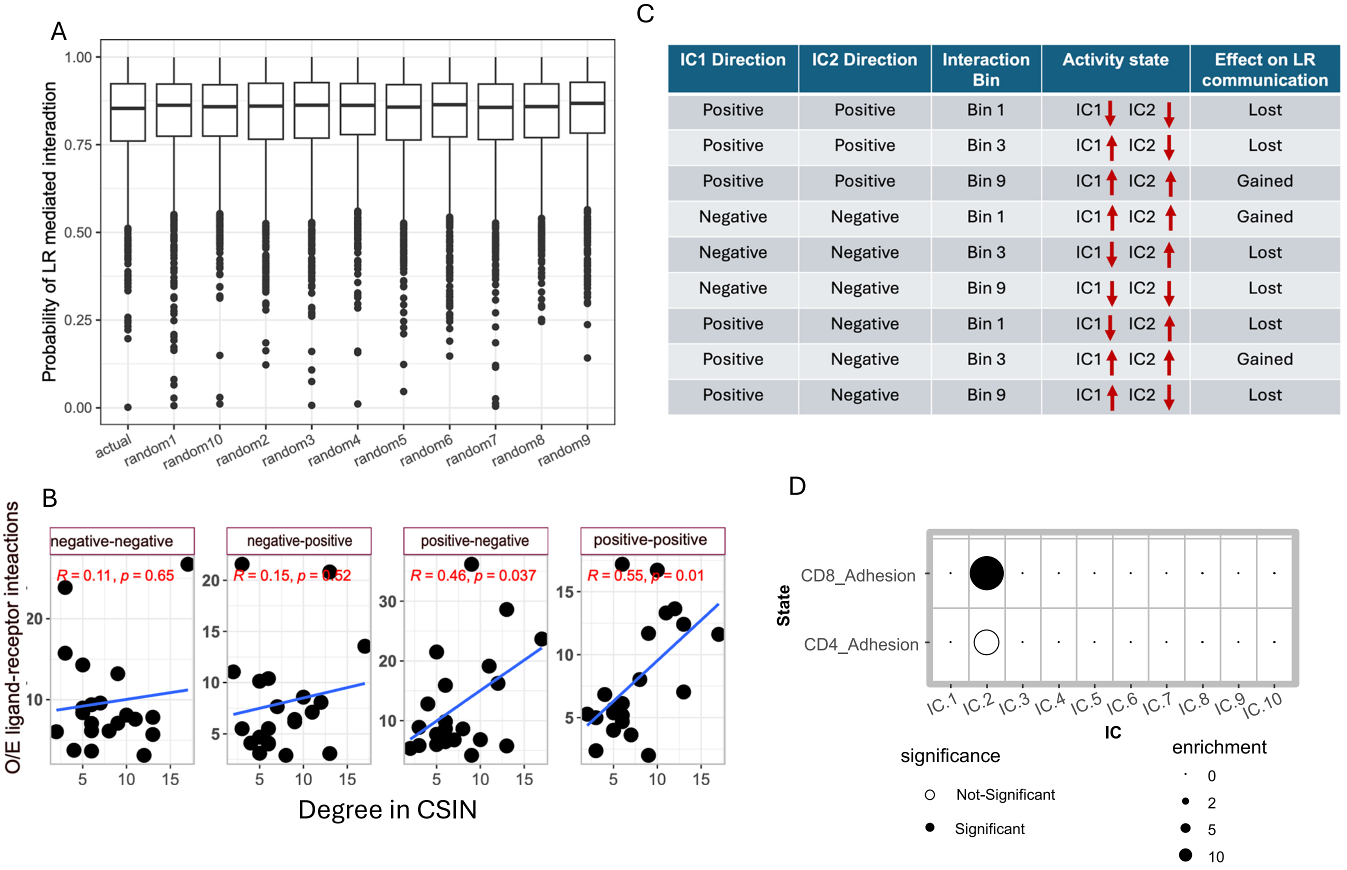

Figure EV4. Analysis of ligand-receptor mediated cell state interactions.

(**A**) Boxplot showing the probability for LR-mediated CSIs among the pairs of ICs in CSIN and 10 randomized networks. There is no statistically significant difference between the inferred CSIN and randomized networks. Samples sizes (N) from left to right are: 902, 1984, 1981, 1982, 1979, 1982, 1982, 1981, 1985, 1983, 1983. The horizontal line in the middle is the median value with lower and upper edges of the box corresponding to the 25th and 75th percentiles and vertical lines corresponding to 1.5 times the interquartile range. (**B**) Scatter plot between the degree of interactions between cell type pairs calculated from CSIN and observed by the expected (O/E) ratio of the complementary ligand receptor pairs present among the signature genes of all IC pairs between two cell types. We perform this calculation by considering all four possible combinations of the positive and negative signature genes of ICs as indicated at the top of each plot. Spearman's correlation coefficient and corresponding *P* value is shown in the plot. (**C**) A schematic table illustrating how the presence of complementary LR pairs among the negative or positive signature genes of ICs affects their activity across different interaction bins. The direction of arrows shows the status of positive and negative signature genes of their corresponding ICs across different interaction bins. A given LR interaction is deemed inactivated if one or both partners are downregulated. (**D**) A dot plot showing the odds ratio for the overlap between the negative signature genes of various T cell ICs and markers of T cell adhesion. Size of dots is proportional to the odds ratio derived from Fisher's exact test and solid points indicate the comparisons where the FDR-corrected *P* value < 0.20. Source data are available online for this figure.

 © This is a U.S. Government work and not under copyright protection in the US; foreign copyright protection may apply

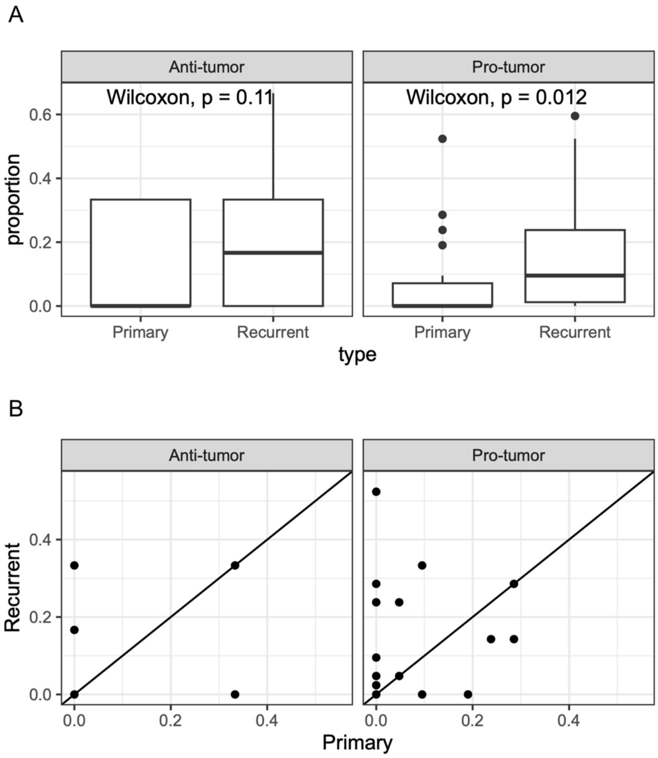

Figure EV5. Association of CSIN with therapy response.

(**A**) Boxplots showing the distributions of CSI load for pro- and anti-tumor interactions among the primary ($N = 31$) and recurrent glioma ($N = 40$) patients. The horizontal line in the middle is the median value, with lower and upper edges of the box corresponding to the 25th and 75th percentiles, and vertical lines corresponding to 1.5 times the interquartile range. (**B**) A scatter plot with the same data as in (**B**). Each dot represents a paired value of interaction load derived from primary (*x*-axis) and recurrent (*y*-axis) tumors from the same patient. Source data are available online for this figure.

© This is a U.S. Government work and not under copyright protection in the US; foreign copyright protection may apply

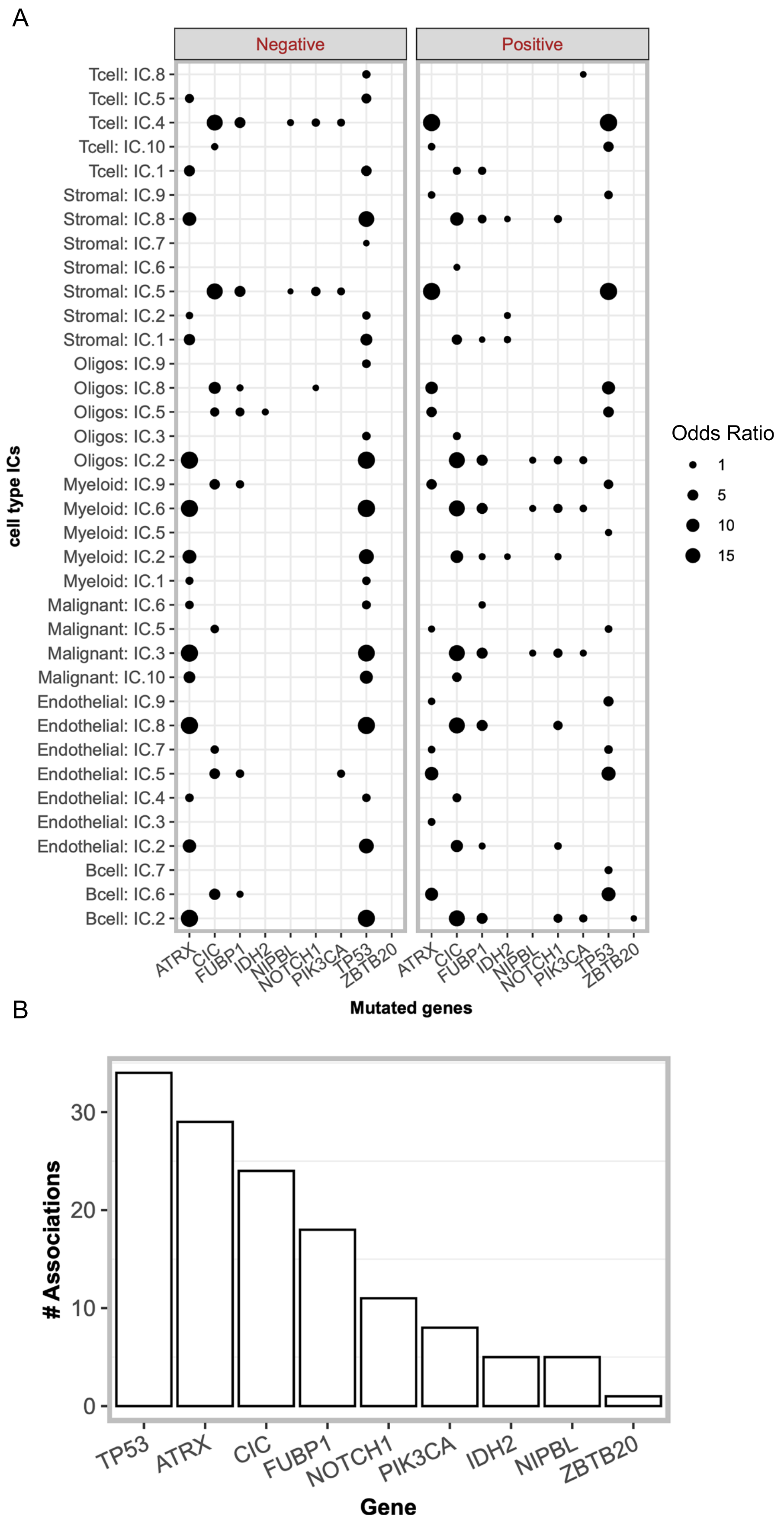

 © This is a U.S. Government work and not under copyright protection in the US; foreign copyright protection may apply

Figure EV6. Association between somatic mutations and CSIN.

(**A**) A dot plot showing the odds ratio for the overlap between IDH-mutant glioma patients having specific nonsynonymous somatic mutations (*x*-axis) and high activity score (~ top 33%) of ICs (*y*-axis). Size of dots is proportional to the odds ratio and solid points indicate Fisher's exact test FDR < 0.20. (**B**) A bar plot showing the number of significantly associated ICs for each significant gene in (**A**). Source data are available online for this figure.

© This is a U.S. Government work and not under copyright protection in the US; foreign copyright protection may apply

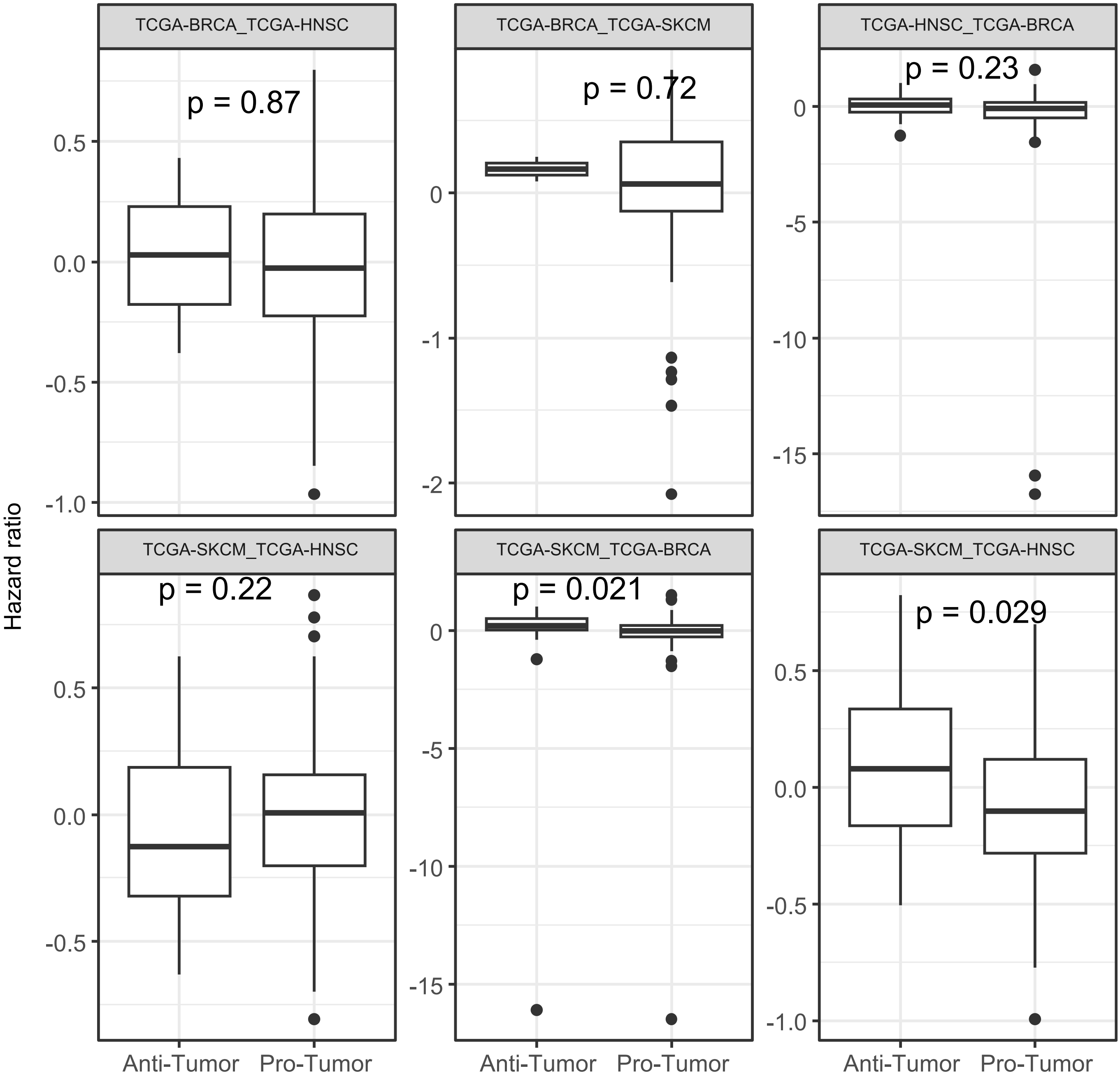

Figure EV7. Cross cancer comparison of cell state interactions.

Boxplots showing the distribution of hazard ratios for pro- and anti-tumor identified in one cancer type (source) and tested in another cancer type (target). The horizontal line in the middle is the median value with lower and upper edges of the box corresponding to the 25th and 75th percentiles and vertical lines corresponding to 1.5 times the interquartile range. Source and target cancer types included in each comparison are indicated at the top of each plot. *P* values are from Wilcoxon's rank-sum test. Source data are available online for this figure.

 © This is a U.S. Government work and not under copyright protection in the US; foreign copyright protection may apply
